# Subcapsular local anesthesia approach in percutaneous liver biopsy: less pain, more comfort

**DOI:** 10.3906/sag-2006-346

**Published:** 2021-02-26

**Authors:** Özgür ÇAKIR, Can AKSU

**Affiliations:** 1 Department of Radiology, Faculty of Medicine, Kocaeli University, Kocaeli Turkey; 2 Department of Anesthesia and Reanimation, Faculty of Medicine, Kocaeli University, Kocaeli Turkey

**Keywords:** Biopsy, liver, pain, local anesthesia

## Abstract

**Background/aim:**

To compare the subjective level of pain in patients who underwent an ultrasound-guided percutaneous liver biopsy (PLB) after either pericapsular anesthesia (PA) or subcapsular anesthesia (SA), based on the numeric rating scale (NRS).

**Materials and methods:**

A total of 323 patients, mean age 51, range 21–82 years; 160 (49.5%) male, referred to the Interventional Radiology Clinic of Kocaeli University Faculty of Medicine for image-guided PLB, between June 2019 and May 2020 were included and randomized into two groups by anesthetic type; the first (n = 171) consisted of patients undergoing SA while the second (n = 152) included patients undergoing PA. The intensity of pain at 0, 1, and 6 h after PLB was evaluated between the groups using NRS.

**Results:**

At hours 0, 1, and 6, the median [range] NRS scores in the subcapsular and pericapsular groups were 2 [1–2] versus 3 [2–4] (P < 0.001), 1 [0–1] versus 1 [1–2] (P < 0.001), and 0 [0–0] versus 1 [0–1] (P < 0.001), respectively. Subgroup analysis revealed that the patients who underwent the subcostal procedure with subcapsular anesthesia reported the lowest pain scores and intercostal procedure with pericapsular anesthesia reported the worst pain scores for each time point: 0 h 1 [1–2] versus 3 [3–4], P < 0.001; 1 h 1 [0–1] versus 1 [1–2], P < 0.001; and 6 h 0 [0–0] versus 0 [0–1], P < 0.001, respectively.

**Conclusion:**

Subcapsular anesthesia is a well-tolerated procedure compared to a pericapsular procedure. Furthermore, the application of a subcapsular anesthetic with a subcostal approach was reported to result in the lowest pain and greatest patient comfort.

## 1. Introduction

Percutaneous liver biopsy (PLB) is a procedure that we obtain a small piece of liver tissue by a needle inserted into the liver through the skin, subcutaneous tissue, and muscles, either with or without the guidance by simultaneous imaging [1,2]. PLB is an essential procedure in assessing chronic liver diseases and the differentiation of primary or metastatic liver malignancies [3,4].

After the biopsy procedure, minor complications such as pain and significant complications such as pneumothorax, hemorrhage, and death may develop [5]. Although pain seems to be a minor complication, it may affect the diagnosis and treatment in liver diseases by affecting the patient’s willingness to have the procedure. Previous studies have demonstrated moderate to severe perception of pain after liver biopsy [2,6,7].

Pain is a sophisticated subjective experience. Pain arising from the liver biopsy is believed to originate from skin and liver capsule innervation [5]. In previous reports, the patient’s age and sex, operator experience, route of biopsy, size of the needle, number of needle passes, and intravenous drug usage have been identified as factors that lead to pain from liver biopsy [8,9].

Traditionally, pericapsular anesthesia is a commonly used approach as a part of liver biopsy [10]. In our institution, two techniques –subcapsular (SA) or pericapsular (PA)– are used for the administration of a local anesthetic. In the past, most of the liver biopsies in our center were performed with PA. However, anecdotal reports from patients indicated that the SA approach was associated with less pain. This study aimed to compare the levels of pain, as reported by patients undergoing PLB, after either SA or PA local anesthesia.

## 2. Materials and methods

### 2.1. Patient selection

The study was designed as a cross-sectional single-center study. All patients who were referred for liver parenchyma biopsy to the Interventional Radiology Clinic of Kocaeli University Faculty of Medicine between June 2019 and May 2020, and met the inclusion criteria were considered for the study. Patients were randomly assigned to one of the local anesthesia approaches, SA or PA.

Adults over 18 years old and with normal coagulation parameters and agreeing to participate in the study were included. Patients under the age of 18 and those with abnormal coagulation test results (international normalized ratio >1.5), low platelet count (<70.000/mm³), dilatation of the biliary ducts, massive ascites, past liver transplantation, and pregnancy were excluded from the study.

### 2.2. Randomization

Patients were randomly assigned into two groups, liver biopsy performed after PA (n = 152) and SA (n = 171). A web-based randomization platform Urbaniak GC, Plous S (2021). Randomizer [online]. Website https://www.randomizer.org/ [accessed 01 July 2020]. was used, and randomization codes for either a PA or SA procedure were placed in sequentially numbered, sealed envelopes in the interventional radiology department. The radiologist who performed the biopsy procedure was aware of the approach, but the patients and the physician (CA) evaluating the pain were blinded to the method.

### 2.3. Procedure

All PLB procedures were guided by ultrasound (US) and were performed by a single experienced operator (OC) with more than ten years’ experience. The operator had achieved more than 100 PLBs using both the subcostal and intercostal approaches before conducting this study. No sedation procedure was administered before the process. 

All the biopsy procedures were performed under an aseptic field, using a 25-gauge needle and a total of 10 mL of 1% lidocaine for anesthesia. In the PA group, 5 mL of 1% lidocaine was injected into subcutaneous tissue and 5 mL of 1% lidocaine to the liver’s periscapular area. In the SA group, 5 mL of 1% lidocaine was injected into the subcutaneous tissue, and the remaining 5 mL of 1% was injected into the liver capsule. 

**Figure 1:**
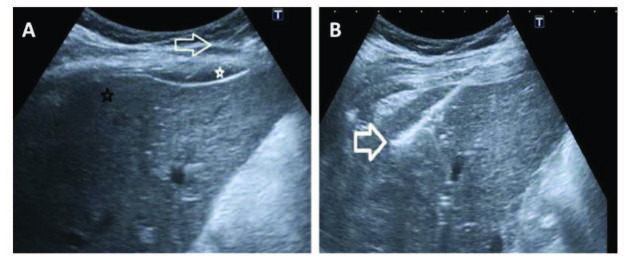
A) US image before the liver biopsy, the needle (white arrow), injected local anaesthetic agent under the liver capsule (white star), liver parenchyma region to be biopsied (black star); B) US image during the liver biopsy. The biopsy needle has been introduced through the anesthetized capsular area (bold white arrow).

Both PA and SA lidocaine injections were performed under US-guidance, and vascular leakage was checked by applying negative aspiration, before giving the local anesthetic agent. The spread of the anesthetic agent delivered to the subcapsular liver area was observed by the US, as shown in Figures 1A and 1B. 

PLB was performed using either an intercostal or subcostal approach as appropriate for the intended biopsy site. We performed the intercostal method from the right lateral lower chest and the subcostal approach from the epigastrium. After a small incision of less than 5 mm, we performed freehand US-guided biopsy with an 18-gauge, automated, cutting, biopsy needle (Magnum; C.R. Bard, Covington, GA, USA). The throw of the cutting needle was set at 22 mm, and if the specimen was judged inadequate in size (≤15 mm in length), we repeated the biopsy procedure at the same access site. After PLB, patients were observed on the ward for 12 h with control of vital signs.

### 2.4. Pain assessment

The numeric rating scale is a scale designed to help assess the extent of an individual’s pain and improve communication regarding pain with health care providers. In our study, we preferred NRS because of our experience in using and evaluating in previous studies in our clinic and ease of application. The level of pain was evaluated with the numerical rating scale (NRS) immediately after the procedure (pain score at 0 h), and after 1 h (pain score at 1 h) and after 6 h (pain score at 6 h). Patients were asked to rate their pain on a 10-point scale, where 0 represents no pain, and 10 represents the worst pain that the patient could imagine. NRS is a validated and reliable tool. The pain scores were subdivided to achieve final ratings of 0–3 (score of 0 = 0; scores of 1–3 = 1; scores of 4–6 = 2; scores of 7–10 = 3) as previously described [11]. 

A physician (CA) informed all the patients about NRS before the biopsy procedure and asked patients to mark the pain levels experienced at 0, 1, and 6 h by guiding them and providing as much information as necessary.

### 2.5. Statistical analysis

Descriptive statistics for demographic characteristics of the patients and features related to the procedure were presented as frequency and percentage (%) for categorical variables and mean with standard deviation (mean ± SD) or median and interquartile range [median (IQR = Q3–Q1)] according to the distribution of the continuous variables. Demographic and clinical features were compared between the subgroups using the Mann–Whitney U test (Wilcoxon rank-sum test), independent sample t-test, or chi-square test as appropriate. We analyzed the relationship between the pain scores and the duration of the procedure by Spearman’s rank correlation coefficient. Kruskal–Wallis H test was used to assess the pain scores between the subgroup analysis of the type of anesthesia and the biopsy location. Dunn’s pairwise tests were carried out with post hoc analysis using Mann–Whitney U test. The p adjusted using the Bonferroni correction.

The sample size was calculated to achieve 80% power to detect 0.6 of a standard deviation difference in NRS with an alpha of 0.05.

All statistical analyses were performed using SPSS, version 20.0 software (IBM Corp., Armonk, NY, USA). Two-sided p values less than 0.05 were considered statistically significant (P < 0.05).

## 3. Results

Final numbers in the PA and SA groups exceeded the power calculation requirement at n = 152 and n = 171, respectively. The comparison of demographics of population and features of procedure between the SA and PA groups is summarized in Table 1.

**Table 1:** Comparison of the demographics of the population and features of the procedures between subcapsular and pericapsular anesthesia.

	Subcapsularn = 171	Pericapsularn = 152	P
Female, n (%)	91 (53.2)	80 (52.6)	0.294
Age, yrs	54 ± 17.3	50.7 ± 16.8	0.089
Biopsy location (subcostal), n (%)	37 (21.6%)	45 (29.6%)	0.101
Pain score at 0. h	2 [1–2]	3 [2–4]	<0.001
Pain score at 1. h	1 [0–1]	1 [1–2]	<0.001
Pain score at 6. h	0 [0–0]	1 [0–1]	<0.001

**Figure 2:**
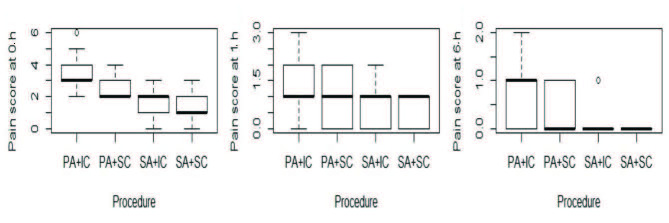
Comparison of the pain scores in the four subgroups: PA+IC pericapsular anesthetic and intercostal approach; PA+SC pericapsular anesthetic and subcostal approach; SA+IC subcapsular anesthetic and intercostal approach; SA+SC subcapsular anesthetic and subcostal approach.

Based on the previous reports suggesting the subcostal approach was superior over intercostal, the pain scores were analyzed after subgrouping for both the anesthesia type and biopsy approach route. It resulted in there being four subgroups: SA with the subcostal procedure (SA+SC), SA with the intercostal procedure (SA+IC), PA with the subcostal procedure (PA-SC), and PA with the intercostal procedure (PA-IC). When the pain scores at 0, 1, and 6 h with subgroups were compared, there was a significant difference in terms of reported pain (Table 2). The pairwise comparison revealed that the PA-IC group reported the worst pain scores while the SA-SC subgroup reported the least pain at all time points, as shown in Figure 2.

**Table 2:** Pain scores in four subgroup comparisons.

	SA+SC	SA+IC	PA+SC	PA+IC	p
Pain score at 0. h	1 [1–2]	2 [1–2]	2 [2–3.5]	3 [3–4]	<0.001
Pain score at 1. h	1 [0–1]	1 [0–1]	1 [0–2]	1 [1–2]	<0.001
Pain score at 6. h	0 [0–0]	0 [0–0]	0 [0–1]	0 [0–1]	<0.001

SA + SC, subcapsular anesthesia + subcostal procedure; SA + IC, subcapsular anesthesia + intercostal procedure; PA + SC, pericapsular anesthesia + subcostal procedure; PA + IC, pericapsular anesthesia + intercostal procedure.

There was no correlation between patient age and pain scores in each anesthesia subgroup (data not shown). There was a significant and positive correlation between the duration of the procedure and pain scores at 0 h in patients who underwent pericapsular anesthesia (Table 3).

**Table 3:** Correlation between the pain scores at 0, 1 and 6 h after the biopsy, and age and duration of the procedure.

	Subcapsular				Pericapsular		
Age	r	95% CI	P		r	95% CI	P
Pain score at 0. h	–0.007	(–0.157)–0.136	0.924		0.044	(–0.105)–0.197	0.570
Pain score at 1. h	–0.036	(–0.189)–0.109	0.636		0.033	(–0.108)–0.172	0.669
Pain score at 6. h	0.093	(–0.059)–0.238	0.226		0.054	(–0.003)–0.116	0.486
Procedure duration	r	95% CI	P		r	95% CI	P
Pain score at 0. h	–0.011	(–0.171)–0.152	0.894		0.210	0.036–0.379	0.010**
Pain score at 1. h	–0.080	(–0.260)–0.077	0.330		-0.008	(–0.175)–0.158	0.921
Pain score at 6. h	–0.105	(–0.267)–0.084	0.197		0.018	(–0.149)–0.192	0.823

## 4. Discussion

Liver biopsy is an essential diagnostic tool for evaluating acute and chronic parenchymal liver diseases as well as mass lesions [1,4]. As a result of therapeutic advances, histological assessment has become a central aspect of diagnosis and staging of the parenchymal liver diseases [12]. Although the typical imaging pattern enables the determination of liver metastasis and hepatocellular carcinoma (HCC) with noninvasive diagnostic techniques (CT or MRI), it can be a significant challenge to diagnose nodules smaller than one centimeter in diameter in cirrhotic liver parenchyma [4,13,14]. If imaging modalities are not sufficient for diagnosis of HCC, histopathological assessment is recommended as a more definitive diagnostic tool but requires invasive procedures such as PLB [15].

Pain is the most common complaint of patients after PLB [2]. It has been reported that 69% of the patients complained about the pain after PLB, with 1.5%–3% requiring hospitalization [16,17]. Pain during and after the liver biopsy is a subjective clinical finding, and it is challenging to address because of its complex nature. The studies have shown a correlation between reported pain and an increased number of biopsies, a close relationship between complications and percutaneous intervention, or biopsy needle thickness [2]. However, the diagnostic accuracy of the histological assessment is closely linked to the number of biopsy samples and the adequacy of samples [18].

We randomized the patients undergoing US-guided PLB into either the SA or the PA groups. Our results showed that the SA approach resulted in lower levels of pain immediately after the procedure, 1 and 6 h postoperatively. Although Pazeshki et al. reported no difference in reported pain between parenchymal PLB and PLB for focal mass lesion, it is not clear if either group of patients received more analgesics before the biopsy procedure because of anxiety [2]. More patients with PA had a history of previous liver biopsy, but a history of previous PLB has not been reported to be associated with higher reported pain in previous studies. Therefore, we suggest that a previous unpleasant biopsy experience may lead to more reported pain in these patients. Interestingly, the procedure duration was longer in the SA group than the PA group, and it may be expected to result in more pain due to several factors. This finding should be investigated further before concluding that a prolonged procedure duration for patients receiving SA may not affect the pain experienced by patients.

We created four subgroups to evaluate the contribution of the intercostal and subcostal routes to the type of local anesthesia; subcapsular anesthesia + subcostal procedure, subcapsular anesthesia + intercostal procedure, pericapsular anesthesia + subcostal procedure and pericapsular anesthesia + intercostal procedure. Consistent with the results of previous reports, we found that the subcostal procedure was associated with less pain in addition to the effect of anesthesia type [2,3,6]. SA with subcostal procedure resulted in the lowest pain scores for all three-time points, and PA with the intercostal procedure was associated with the worst NRS scores. Our study is the first, in terms of pain assessment, to investigate the combination of route and anesthesia type in PLB. We found no correlation between procedure duration and pain in the SA group. However, there was a weak but significant positive correlation between reported pain immediately after the biopsy and duration in the PA group. Age did not correlate with pain at three different points of evaluation in both groups. 

One of our study’s main finding was a significant difference between the pain scores in favour of the subcapsular anesthesia procedure. The critical point is that patients with hepatitis are usually young, asymptomatic, and need repeated biopsies for management [12]. Unfortunately, unpleasant experiences at the first biopsy may result in patients being lost to follow up and, thus, suboptimal treatment results. The second main finding was that the subcapsular anesthesia/subcostal approach combination resulted in significantly better pain scores. Although previous studies have evaluated many factors, our study is the first to evaluate the biopsy route and anesthesia technique together [2,3,7].

A significant limitation of this study was that we did not assess the level of anxiety before and after the biopsy procedure. Anxiety level was associated with pain levels and also correlated with an increase in analgesia use after biopsy [5, 19]. There is a need for studies that evaluate pre-procedure anxiety and the effect of either SA or PA in PLB. Another limitation is the heterogeneity of the two anesthesia groups in terms of several factors, including needle type, needle thickness, biopsy indication, which may be confounding for pain, and homogenization of the groups may result in different results.

In summary, the results of our study suggest that subcapsular anesthesia is superior to pericapsular anesthesia. The use of the combination of SA and subcostal liver biopsy route seems to be the best approach among the four subgroups and resulted in the lowest pain scores. Improving the patients’ experience of PLB by the type of anesthesia and route of intervention may ameliorate the negative effect of previous intolerable experiences for patients and result in a greater willingness to undergo further biopsies.

## Informed consent

The study was approved by the local ethics committee (Kocaeli University Faculty of Medicine Ethics Committee, Kocaeli, Turkey), as stated in the protocol of research number (GOKAEK 2019/229). All patients participating in the study were provided with detailed information, and informed consent was obtained for the study from all study participants in addition to consent routinely obtained for the procedure.
